# From Periodontal Inflammation to Atrial Fibrillation: Molecular Links Through Thrombospondin-1, Galectin-3, and SGLT2-Related Pathways

**DOI:** 10.3390/ijms27188076

**Published:** 2026-09-11

**Authors:** Violeta Ariana Nicoras, Daniel Florin Lighezan, Horia Silviu Branea, Andreea Munteanu, Doina Georgescu, Adrian Sebastian Zus, Diana Alexandra Mitu, Romina Georgiana Bita

**Affiliations:** 1 Doctoral School, “Victor Babes” University of Medicine and Pharmacy, 300041 Timisoara, Romania; ariana.nicoras@umft.ro; 2 Center for Advanced Research in Cardiovascular Pathology and Hemostaseology, 1st Internal Medicine Department, 1st Medical Semiology Discipline, “Victor Babes” University of Medicine and Pharmacy, 300041 Timisoara, Romania; dlighezan@umft.ro (D.F.L.); doina.georgescu@umft.ro (D.G.); diana-alexandra.mitu@umft.ro (D.A.M.); 3 Department of Internal Medicine I—Medical Semiotics II, “Victor Babes” University of Medicine and Pharmacy, Eftimie Murgu Sq. No. 2, 300041 Timisoara, Romania; 4 Internal Medicine I—Discipline of Internal Medicine IV, Department V, Center of Advanced Research in Cardiology and Hemostaseology, “Victor Babes” University of Medicine and Pharmacy, Eftimie Murgu Sq. No. 2, 300041 Timisoara, Romania; 5 Department VI—Cardiology, “Victor Babes ” University of Medicine and Pharmacy, Eftimie Murgu Sq. No. 2, 300041 Timișoara, Romania; adrian.zus@umft.ro; 6 2nd Department, Radiology and Medical Imaging, General and Dento-Maxillary Imaging, Dental Medicine Faculty, “Victor Babes” University of Medicine and Pharmacy, 300041 Timisoara, Romania; romina.bita@umft.ro

**Keywords:** periodontitis, systemic inflammation, endothelial dysfunction, atrial fibrosis, atrial fibrillation, thrombospondin-1, galectin-3, SGLT2

## Abstract

Periodontitis is a chronic inflammatory disease initiated by dysbiotic subgingival biofilms and characterized by local tissue destruction, epithelial ulceration, immune-cell activation, and extracellular-matrix remodeling. Increasing evidence suggests that periodontal inflammation may extend beyond the oral cavity through recurrent bacteremia, dissemination of microbial products, systemic cytokine activation, endothelial dysfunction, oxidative stress, and thrombo-inflammatory signaling. These mechanisms are also implicated in atrial structural remodeling and atrial fibrillation (AF), but the molecular bridge between periodontal lesions, systemic inflammatory–fibrotic activity, and the atrial substrate remains incompletely defined. This structured narrative review synthesizes mechanistic, translational, biomarker-based, observational, and interventional evidence linking periodontal inflammation with cardiovascular remodeling and AF. Literature was identified through searches of PubMed/MEDLINE, Scopus, and Web of Science, complemented by reference-list screening. Particular attention was given to thrombospondin-1 (TSP-1), transforming growth factor-β (TGF-β)-related signaling, galectin-3 (Gal-3), and SGLT2-related cardiometabolic pathways. Because of heterogeneity in study populations, periodontal definitions, biomarker measurements, interventions, and cardiovascular endpoints, findings were synthesized thematically rather than quantitatively. Periodontitis is associated with systemic inflammatory activation, endothelial dysfunction, oxidative stress, and cardiovascular phenotypes, including atherosclerotic disease, hypertension, heart failure, and AF. Epidemiological studies support an association between periodontitis and incident AF, while human tissue data link periodontal inflammatory burden with atrial fibrosis, non-paroxysmal AF, and left atrial appendage thrombosis in selected AF populations. The strength of evidence differs across the proposed pathways: Gal-3 has the most consistent clinical evidence in AF and cardiac fibrosis; TSP-1/TGF-ß signaling is supported mainly by mechanistic periodontal-tissue and selected cardiovascular studies; and SGLT2-related pathways remain indirect systemic modifiers of the cardiometabolic and inflammatory milieu. Evidence regarding periodontal therapy and AF-related outcomes remains promising but limited and does not establish a direct antiarrhythmic effect. The available evidence supports a biologically plausible inflammatory–fibrotic continuum linking periodontitis, systemic immune activation, endothelial dysfunction, atrial remodeling, and AF susceptibility. However, causality, temporal sequence, tissue-level concordance, and biomarker-based clinical utility remain insufficiently established. Future studies integrating standardized periodontal assessment, circulating biomarkers, tissue-level inflammatory–fibrotic markers, cardiac imaging, and rhythm monitoring are needed to clarify whether local periodontal molecular signatures reflect systemic or cardiac remodeling relevant to AF vulnerability.

## 1. Introduction

Chronic inflammation contributes to both cardiovascular diseases and periodontitis. Periodontitis is initiated by dysbiotic subgingival biofilms and sustained by local immune activation, cytokine release, epithelial ulceration, and extracellular-matrix remodeling. Because inflamed periodontal pockets can allow recurrent bacteremia and dissemination of microbial products, periodontal disease may also contribute to a low-grade systemic inflammatory state relevant to vascular dysfunction and cardiovascular risk [1,2,3].

Epidemiological and mechanistic studies have associated periodontitis with atherosclerotic cardiovascular disease and vascular inflammation, although residual confounding by age, smoking, diabetes, obesity, socioeconomic status, and access to care remains important [3]. The relevance of this association for the present review is not the general cardiovascular risk alone, but the inflammatory, endothelial, thrombo-inflammatory, and fibrotic background through which chronic oral inflammation may interact with atrial remodeling.

Systemic inflammation is also implicated in atrial fibrillation (AF). Pro-inflammatory mediators can promote atrial fibroblast activation, collagen deposition, endothelial dysfunction, and conduction heterogeneity, thereby contributing to the atrial substrate required for AF initiation and maintenance [4,5]. This supports the view that AF should be interpreted not only as an electrical disorder but also as part of an atrial cardiomyopathy phenotype influenced by inflammatory and fibrotic remodeling.

On this basis, chronic periodontal inflammation has been proposed as a potential contributor to AF vulnerability. In a nationwide cohort of more than 1.25 million individuals followed for approximately 14 years, persistent periodontitis was associated with a higher incidence of AF, while improvement of periodontal status was associated with a slightly lower risk than persistent disease [6].

Interventional evidence is more limited but clinically relevant. In patients undergoing AF ablation, periodontal treatment during the blanking period was associated with lower AF recurrence, particularly in those with severe periodontitis [7]. These findings are supportive but should not be interpreted as proof that periodontal treatment has a direct antiarrhythmic effect.

Recent studies further support the periodontitis–AF association. A 2025 synthesis reported a dose-dependent relationship between periodontitis severity and AF risk and highlighted possible shared immune-genetic pathways [8]. Complementary mechanistic work demonstrated that *Porphyromonas gingivalis* may translocate from periodontal tissue to the atrial myocardium and promote atrial fibrosis through Gal-3 and TGF-β1 signaling in experimental models, providing important but still early tissue-level support [9].

At the molecular level, this review focuses on three pathways with different levels of evidence and different biological meanings. Galectin-3 (Gal-3) has the strongest clinical evidence in AF and cardiac fibrosis. Thrombospondin-1 (TSP-1)/TGF-β signaling is relevant mainly as a mechanistic and tissue-level inflammatory–fibrotic pathway. SGLT2-related pathways should be considered separately, as potential systemic cardiometabolic modifiers rather than direct periodontal or atrial tissue biomarkers. This hierarchy is important because the available data do not demonstrate all components of the proposed pathway with the same strength [10,11,12,13,14,15].

Beyond local oral pathology, periodontal inflammation should therefore be placed within a broader cardiometabolic and inflammatory–fibrotic context. Metabolic stress, insulin resistance, endothelial dysfunction, systemic inflammation, and fibrotic biomarker activation may all shape AF vulnerability, but these factors should be interpreted as background modifiers rather than direct proof of a periodontitis-driven AF mechanism [16,17,18,19,20].

**Figure 1:**
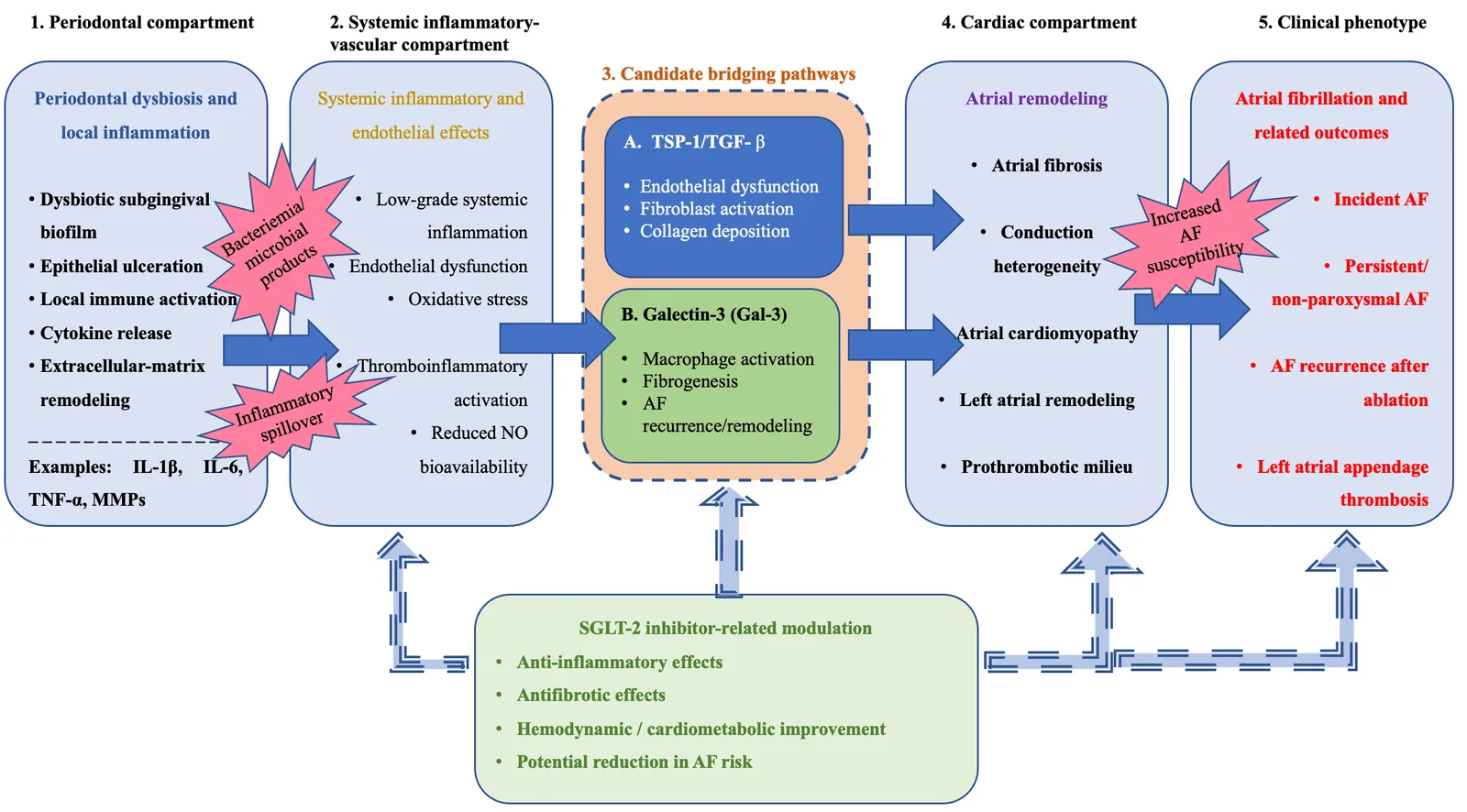
Proposed inflammatory-fibrotic continuum linking periodontitis with atrial remodeling and atrial fibrillation. Periodontal dysbiosis and chronic local inflammation may promote recurrent bacteremia, dissemination of microbial products, and systemic cytokine activation. This inflammatory spillover may contribute to endothelial dysfunction, oxidative stress, thrombo-inflammatory activation, and profibrotic signaling. Among candidate molecular mediators, thrombospondin-1 (TSP-1)/transforming growth factor-β (TGF-β) signaling and galectin-3 (Gal-3) may bridge periodontal inflammatory activity and atrial fibrotic remodeling. These mechanisms may favor atrial cardiomyopathy, conduction heterogeneity, and increased susceptibility to atrial fibrillation. SGLT2 inhibitor-related pathways are shown as indirect systemic modifiers that may attenuate inflammation, fibrosis, and AF risk. Solid arrows indicate better-supported clinical or tissue-level relationships; dashed arrows indicate indirect, extrapolated, or hypothesis-generating links.

Despite increasing epidemiological signals, the periodontitis–AF relationship remains incompletely defined in terms of causality, the relative contribution of atrial “inflamofibrosis,” and the clinical readiness of biomarker-based risk stratification. Furthermore, the translational bridge between oral inflammation and atrial substrate—particularly human tissue-level evidence—remains limited. In this review, we focus on thrombospondins (TSPs), Gal-3, and the SGLT2 axis because they sit at the interface of inflammation–fibrosis biology and have emerging relevance as measurable biomarkers and/or actionable therapeutic pathways (Figure 1). Other inflammatory mechanisms (e.g., IL-1β/NLRP3 signaling, NET-related pathways) are acknowledged where relevant, but are not the primary focus of this synthesis.

The objective of this review is to synthesize mechanistic, tissue-level, biomarker-based, and clinical evidence linking periodontal inflammation with atrial remodeling and atrial fibrillation, with particular emphasis on TSP-1/TGF-β-related signaling, Gal-3, and SGLT2-related cardiometabolic modulation. By integrating periodontal, systemic, and cardiac evidence, this review aims to clarify candidate inflammatory–fibrotic pathways, highlight unresolved mechanistic questions, and support a more interdisciplinary interpretation of cardiovascular risk in patients with chronic periodontal inflammation.

## 2. Methods

This article was designed as a structured narrative review that integrates mechanistic, translational, and clinical evidence on the relationship among chronic periodontal inflammation, cardiovascular remodeling, and atrial fibrillation. Particular attention was given to inflammatory and profibrotic pathways involving thrombospondins, galectin-3, and SGLT2-related cardiometabolic mechanisms. Evidence concerning atherosclerosis, endothelial dysfunction, hypertension, heart failure, and cerebrovascular risk was included when it contributed to understanding the broader inflammatory–fibrotic continuum relevant to atrial remodeling. Because the available literature encompasses heterogeneous experimental models, biomarker studies, observational cohorts, interventional investigations, and prior systematic reviews, a narrative synthesis was considered more appropriate than quantitative pooling of evidence. A structured literature search was conducted in PubMed/MEDLINE, Scopus, and Web of Science between 1 October 2025 and 5 January 2026. The search covered publications from January 2000 through December 2025, with preference given to more recent evidence when studies had comparable relevance and methodological quality. A targeted update for directly relevant cardiometabolic and atrial fibrillation literature was subsequently performed through February 2026. Search terms were combined using Boolean operators and included terms related to periodontal disease, cardiovascular disease, inflammation, atrial remodeling, and molecular biomarkers. Principal terms included “periodontitis,” “periodontal disease,” “periodontal inflammation,” “atrial fibrillation,” “atrial remodeling,” “atrial fibrosis,” “atherosclerosis,” “cardiovascular disease,” “endothelial dysfunction,” “systemic inflammation,” “oxidative stress,” “CRP,” “IL-6,” “TNF-α,” “TGF-β,” “NLRP3,” “thrombospondin,” “galectin-3,” and “SGLT2 inhibitors.” Relevant microbiological terms, including “*Porphyromonas gingivalis*,” “*Aggregatibacter actinomycetemcomitans*,” “bacteremia,” and “lipopolysaccharide,” were also used. Database searching was complemented by examination of the reference lists of key original studies, consensus documents, narrative reviews, systematic reviews, and meta-analyses.

Representative search strategies included: (periodontitis OR periodontal disease OR periodontal inflammation) AND (atrial fibrillation OR atrial remodeling OR atrial fibrosis); (periodontitis OR *Porphyromonas gingivalis* OR lipopolysaccharide) AND (systemic inflammation OR endothelial dysfunction OR oxidative stress OR cytokines); (thrombospondin-1 OR TSP-1 OR TGF-β) AND (periodontitis OR fibrosis OR atrial fibrillation); (galectin-3 OR Gal-3) AND (periodontitis OR myocardial fibrosis OR atrial fibrillation OR ablation recurrence); and (SGLT2 inhibitor OR sodium–glucose cotransporter 2) AND (atrial fibrillation OR inflammation OR fibrosis OR diabetes OR heart failure). Searches were adapted to the syntax of each database, and reference lists of highly relevant articles were screened to identify additional mechanistic, translational, and clinical studies.

Evidence was selected according to its relevance to the principal objectives of the review. Priority was given to:

- Human observational studies examining associations between periodontitis, cardiovascular disease, atrial remodeling, or atrial fibrillation;
- Interventional studies evaluating periodontal treatment or relevant cardiometabolic therapies;
- Studies investigating circulating or tissue biomarkers associated with inflammation and fibrosis;
- Experimental studies providing mechanistic evidence regarding bacterial translocation, immune activation, endothelial dysfunction, oxidative stress, or fibrotic remodeling;
- Systematic reviews, meta-analyses, and consensus statements providing broader contextual evidence.

Case reports were generally excluded, except where they illustrated a potentially relevant but insufficiently studied therapeutic or mechanistic observation. Studies with inadequately defined periodontal exposure, unclear cardiovascular outcomes, or limited relevance to the review’s mechanistic framework were not prioritized. The selection process was purposive rather than exhaustive and was intended to identify the most relevant evidence required for a critical and interdisciplinary narrative synthesis.

The evidence was synthesized thematically across the following domains:

1. Periodontal dysbiosis, bacteremia, and systemic inflammatory activation;
2. Endothelial dysfunction, oxidative stress, and thromboinflammation;
3. Inflammatory and profibrotic mechanisms contributing to atrial remodeling;
4. The potential roles of thrombospondins and galectin-3 as molecular mediators and biomarkers;
5. The indirect anti-inflammatory, antifibrotic, and cardiometabolic effects of SGLT2 inhibitors;
6. Clinical evidence linking periodontitis with atrial fibrillation incidence and recurrence;
7. Limitations of the current evidence and unresolved mechanistic questions.

Because of substantial heterogeneity in study populations, periodontal definitions, biomarker measurements, interventions, and cardiovascular endpoints, a quantitative meta-analysis was not performed. No formal protocol registration or standardized risk-of-bias assessment was performed, as the article was conceived as a structured narrative review rather than a systematic review. Findings derived from small studies, indirect comparisons, experimental models, or secondary analyses were interpreted as hypothesis-generating.

## 3. Results

### 3.1. Periodontitis as a Local and Systemic Inflammatory Disease

Periodontitis is a chronic, multifactorial inflammatory disease initiated by dysbiotic subgingival biofilms and characterized by progressive destruction of the tooth-supporting tissues. Its principal clinical manifestations include periodontal attachment loss, alveolar bone resorption, periodontal-pocket formation, and gingival bleeding on probing. According to the 2017 World Workshop classification, periodontitis is characterized by stage, which reflects disease severity and management complexity, and grade, which estimates the rate of progression and the influence of modifying factors such as smoking and diabetes [21]. In the context of cardiovascular disease, the relevance of this classification lies primarily in its capacity to characterize the extent, activity, and persistence of the periodontal inflammatory burden.

Periodontal pockets contain a high microbial load, including pathogens such as *Porphyromonas gingivalis* and *Aggregatibacter actinomycetemcomitans*. Ulceration of the periodontal-pocket epithelium facilitates episodic entry of bacteria, lipopolysaccharides, and other microbial products into the circulation, resulting in transient bacteremia and potential hematogenous dissemination to distant tissues [22]. Periodontal pathogens or their molecular components have also been identified within vascular lesions, supporting a biologically plausible connection between chronic oral infection and vascular inflammation [23]. In parallel with microbial dissemination, the local immune response within periodontal tissue contributes to systemic inflammatory activation. Periodontal lesions release pro-inflammatory mediators, including IL-1β, IL-6, TNF-α, prostaglandin E2, and matrix metalloproteinases. These mediators may enter the circulation and stimulate the hepatic acute-phase response, thereby contributing to increased circulating concentrations of C-reactive protein (CRP) and other inflammatory markers in patients with active periodontitis compared with periodontally healthy individuals. These persistently circulating pro-inflammatory mediators induce a state of low-grade chronic inflammation in the host, characterized by a moderate (~2–3-fold) increase in cytokine and acute-phase protein levels (as opposed to the hundreds-fold increases seen in acute infections). The chronic systemic inflammation generated in this way can activate pro-inflammatory pathways in multiple organs and tissues; for example, at the level of the vascular endothelium, it may cause endothelial dysfunction and a pro-coagulant state, thereby promoting the development of cardiovascular pathology [24].

Repeated exposure to periodontal microbial products and inflammatory mediators may also induce sustained activation of circulating monocytes and neutrophils. This phenomenon has been interpreted as a form of trained innate immunity, in which prior inflammatory stimulation enhances subsequent immune responses and contributes to a persistent systemic inflammatory state [21,24]. Consequently, periodontitis may act not only as a localized oral disease but also as a chronic source of low-grade systemic inflammation.

These mechanisms indicate that periodontal lesions represent both a source of systemic inflammatory signals and a local tissue environment in which microbial activation, immune-cell recruitment, cytokine signaling, and extracellular-matrix remodeling occur simultaneously. The resulting inflammatory burden may contribute to endothelial dysfunction, oxidative stress, prothrombotic activation, and profibrotic signaling, thereby providing a mechanistic link between periodontal disease and cardiovascular remodeling.

### 3.2. Endothelial Dysfunction, Oxidative Stress, and Cardiovascular Remodeling

Numerous clinical and epidemiological studies over the last decade have investigated the relationship between periodontitis and cardiovascular diseases. Chronic periodontal inflammation has been associated with several cardiovascular phenotypes, including atherosclerotic cardiovascular disease, arterial hypertension, and heart failure. Although these conditions differ clinically, they share biological mechanisms involving persistent systemic inflammation, endothelial dysfunction, oxidative stress, thrombo-inflammatory activation, and progressive tissue remodeling.

Periodontitis has been consistently associated with atherosclerotic cardiovascular disease, including coronary artery disease, myocardial infarction, and stroke. Observational evidence indicates that this association persists after adjustment for several conventional cardiovascular risk factors, although residual confounding and shared determinants such as smoking, diabetes, age, and socioeconomic status remain important considerations [3]. Circulating concentrations of CRP and IL-6 are frequently increased in patients with active periodontitis, while periodontal bacterial components, including DNA derived from *P. gingivalis*, have been identified in atherosclerotic plaques [21]. These findings support biological plausibility but do not, by themselves, establish a direct causal role for periodontal pathogens in atherogenesis. Systemic inflammatory mediators released or amplified in the setting of periodontitis may promote endothelial activation and impair vascular homeostasis. Increased cytokine signaling and oxidative stress may reduce nitric oxide bioavailability, favor leukocyte adhesion and vascular permeability, and contribute to a procoagulant environment. In this context, intensive periodontal treatment has been associated with reductions in circulating inflammatory markers and improvement in some measures of endothelial function, suggesting that at least part of the vascular dysfunction accompanying periodontitis may be modifiable [24]. However, the durability of these effects and their influence on major cardiovascular outcomes remain uncertain.

A similar inflammatory–vascular framework may contribute to the association between periodontitis and arterial hypertension. Periodontal disease has been linked with higher blood-pressure values, more difficult blood-pressure control, and modest blood-pressure improvement after periodontal therapy in some studies [25]. These findings support a shared inflammatory and endothelial background, but they remain heterogeneous and should be interpreted as contextual cardiovascular evidence rather than as direct proof of a periodontitis–AF mechanism.

Evidence linking periodontitis with heart failure is less extensive but is compatible with the same systemic model. Persistent cytokine activation, endothelial dysfunction, and oxidative stress may contribute to myocardial extracellular-matrix remodeling, interstitial fibrosis, ventricular stiffness, and impaired cardiac function. Population-based observations have identified periodontitis as a marker of increased heart-failure risk after adjustment for several confounding variables; nevertheless, these findings remain observational and should not be interpreted as proof that periodontal inflammation independently causes heart failure [26].

Collectively, these cardiovascular associations indicate that periodontitis may coexist with, and potentially amplify, a systemic environment characterized by vascular dysfunction, inflammatory activation, thrombogenicity, and fibrotic remodeling. These mechanisms are not specific to atrial fibrillation, but they provide a biologically coherent background through which persistent periodontal inflammation could contribute to the development of an atrial substrate vulnerable to structural and electrical remodeling.

### 3.3. Clinical and Histological Evidence Linking Periodontitis to Atrial Fibrillation

Epidemiological studies have reported an association between periodontal disease and atrial fibrillation. In a large population-based cohort of more than 1.25 million individuals without AF at baseline, followed for approximately 14 years, persistent periodontitis was associated with a higher incidence of AF than the absence of periodontal disease. Participants whose periodontal disease improved during follow-up had a slightly lower AF risk than those with persistently active disease, suggesting that the duration and persistence of periodontal inflammation may influence the observed association [6].

Additional evidence has been provided by the Atherosclerosis Risk in Communities study, in which severe periodontitis was associated with an increased risk of incident AF compared with good periodontal health [27]. A subsequent meta-analysis also reported a higher combined risk of AF and atrial flutter among individuals with periodontal disease [28]. Although these findings were generally maintained after adjustment for several conventional cardiovascular risk factors, residual confounding remains possible because periodontitis and AF share important determinants, including age, smoking, diabetes, obesity, socioeconomic status, and limited access to preventive healthcare.

The epidemiological association is biologically compatible with the systemic effects of periodontal inflammation. Recurrent exposure to periodontal microbial products and pro-inflammatory mediators may contribute to circulating cytokine activation, oxidative stress, endothelial dysfunction, and profibrotic signaling. These processes may promote atrial extracellular-matrix remodeling, conduction heterogeneity, and increased susceptibility to the initiation and maintenance of AF. Nevertheless, observational associations cannot determine whether periodontitis contributes directly to AF development, acts as a marker of cumulative inflammatory burden, or reflects the influence of shared cardiometabolic risk factors.

Human tissue studies provide a more direct connection between periodontal inflammatory burden and the atrial substrate. In a Japanese study including 76 patients with AF who underwent surgical excision of the left atrial appendage, periodontal indices, including gingival bleeding, periodontal-pocket depth, and the periodontal inflamed surface area, were associated with the extent of histologically assessed atrial fibrosis [29,30]. Patients with greater periodontal inflammatory burden showed more extensive interstitial fibrotic remodeling in atrial tissue, supporting a potential relationship between active periodontal inflammation and the structural substrate that sustains AF.

Greater periodontal inflammatory surface area has also been associated with non-paroxysmal AF and the presence of left atrial appendage thrombus [29]. These findings suggest that periodontal inflammatory burden may be related not only to atrial fibrotic remodeling but also to the prothrombotic environment accompanying more advanced AF phenotypes. However, because these observations were obtained in selected populations with established AF and surgical indications, they cannot establish temporal sequence or causality.

A 2025 systematic review confirmed a consistent positive association between periodontal disease and incident AF across available cohort studies, reinforcing the epidemiological findings summarized above [31]. More directly relevant to mechanistic plausibility, a 2025 study used 16S rRNA gene sequencing to detect *Porphyromonas gingivalis* DNA within the atrial appendages of AF patients and, in a complementary murine model, showed that this bacterial translocation actively promoted atrial fibrosis through upregulation of galectin-3 and TGF-β1 signaling-directly linking a specific periodontal pathogen to the fibrotic pathways discussed further in Section 3.4 and Section 3.5 [9]. Emerging genetic evidence further supports a shared immunoinflammatory basis for the periodontitis-AF association: a bioinformatic analysis of transcriptomic and immune-cell profiling data identified 21 genes common to both conditions, with the Regulator of G-protein Signaling 1 (RGS1) gene emerging as a central node; experimental induction of periodontitis in rats reproduced this signature, with increased atrial fibrosis, greater AF susceptibility, and upregulated cardiac RGS1 expression [32].

Collectively, epidemiological and human tissue evidence supports an association between periodontitis, atrial remodeling, and AF. The histological findings are particularly relevant because they connect clinically quantified periodontal inflammation with structural alterations in cardiac tissue. However, the molecular pathways linking the periodontal lesion to the atrial substrate remain incompletely characterized, and the extent to which local periodontal molecular changes parallel systemic or atrial inflammatory–fibrotic activity is still uncertain.

### 3.4. Thrombospondin-1 and TGF-β-Related Signaling

Thrombospondins are matricellular glycoproteins involved in the regulation of cell–cell and cell–matrix interactions, with important roles in inflammation, angiogenesis, endothelial function, and tissue remodeling. Among this family, thrombospondin-1 (TSP-1) is particularly relevant to the periodontal–cardiovascular interface because it connects immune-cell activation with endothelial dysfunction and profibrotic signaling. TSP-1 can modulate macrophage chemotaxis, neutrophil phagocytosis, and T-cell activation through CD47-dependent mechanisms [33,34]. It also exerts anti-angiogenic effects by interacting with endothelial receptors such as CD36 and CD47, thereby inhibiting VEGF-mediated signaling and reducing nitric oxide bioavailability [33,35]. Through these mechanisms, TSP-1 may contribute to endothelial dysfunction, impaired vascular repair, leukocyte adhesion, and platelet activation [33,35].

A central mechanism linking TSP-1 with fibrotic remodeling is its capacity to activate latent transforming growth factor-β (TGF-β). By facilitating TGF-β activation, TSP-1 promotes fibroblast activation, myofibroblast differentiation, and collagen deposition in chronically inflamed tissues [33,35]. These mechanisms are relevant not only in periodontal lesions, where extracellular-matrix remodeling contributes to tissue destruction and repair, but also in cardiovascular remodeling, where excessive collagen deposition may promote myocardial stiffness, atrial fibrosis, conduction heterogeneity, and maintenance of atrial fibrillation.

In periodontal inflammation, TSP-1 appears to be locally upregulated. Inflamed gingival tissue from patients with periodontitis has been reported to show increased TSP-1 expression compared with healthy gingiva. Experimental evidence further supports this association: *Porphyromonas gingivalis* lipopolysaccharide can stimulate TSP-1 production in monocytic cells through Toll-like receptor activation, NF-κB signaling, and amplification by pro-inflammatory cytokines such as IL-17F [10]. In this context, local TSP-1 overexpression may contribute to osteoclast activation, alveolar bone resorption, inflammatory amplification, and extracellular-matrix remodeling within periodontal lesions [10,36].

Clinical periodontal data also suggest activation of the local TSP-1 axis. Gingival crevicular fluid studies have reported higher TSP-1 concentrations in patients with periodontitis compared with periodontally healthy individuals. Moreover, the coexistence of periodontitis with systemic inflammatory conditions such as non-alcoholic fatty liver disease has been associated with even higher gingival crevicular fluid TSP-1 concentrations, supporting the concept that local periodontal inflammation and systemic inflammatory burden may interact [36]. These findings indicate that periodontal tissues may represent an accessible inflammatory–fibrotic compartment in which TSP-1-related signaling is locally expressed.

In the cardiovascular context, TSP-1 has been implicated in endothelial dysfunction and myocardial fibrotic remodeling. Increased TSP-1 activity may reduce nitric oxide bioavailability and favor vasoconstriction, leukocyte adhesion, and platelet activation, while TSP-1-mediated TGF-β activation provides a plausible pathway toward interstitial fibrosis [33,35]. TGF-β, and matrix-remodeling markers have also been correlated with AF in patients without structural heart disease [34]. In selected cardiovascular literature, the TSP-1/TGF-β/MMP-9 axis has been linked with atrial fibrosis in AF-related settings [35].

Additional clinical evidence supports the association between TSP-1 and atrial arrhythmic vulnerability. In patients with acute myocardial infarction, those who developed atrial arrhythmias, including atrial fibrillation or atrial flutter, had higher plasma TSP-1 concentrations than those without atrial arrhythmias. TSP-1 was also identified as an independent predictor of post-infarction atrial fibrillation or atrial flutter [37]. Although this evidence was not derived from patients selected for periodontitis, it supports the broader concept that increased TSP-1 may reflect a pro-inflammatory and profibrotic milieu capable of facilitating atrial electrical and structural remodeling.

Overall, TSP-1 remains best interpreted as a plausible mechanistic bridge between periodontal inflammation, endothelial dysfunction, and atrial fibrotic remodeling. Its local upregulation in periodontal inflammatory environments and its involvement in TGF-β-related cardiac fibrosis support the concept of a shared inflammatory–fibrotic pathway. However, direct evidence demonstrating concordance between periodontal-tissue TSP-1 expression, circulating TSP-1 concentrations, and atrial remodeling severity remains limited. Therefore, TSP-1 should currently be regarded as a biologically plausible bridging marker rather than an established causal mediator of the periodontitis–AF relationship.

### 3.5. Galectin-3 and the Inflammatory–Fibrotic Continuum

Galectins are soluble lectin proteins that bind β-galactoside-containing glycoconjugates on cell-surface and extracellular-matrix proteins. Among them, galectin-3 (Gal-3) is particularly relevant to cardiometabolic and inflammatory disease because it participates in immune-cell activation, macrophage polarization, fibroblast stimulation, and extracellular-matrix remodeling. In inflammatory tissues, Gal-3 may function as a mediator of immune amplification, while in chronic remodeling states, it contributes to a profibrotic environment by promoting fibroblast proliferation, collagen deposition, and stabilization of extracellular-matrix components [38,39].

In periodontal tissues, Gal-3 has been implicated in the local inflammatory response. It modulates neutrophil, macrophage, and T-cell activity and may influence the interaction between host immune responses and the local microbial environment [38]. Clinical studies have reported increased Gal-3 concentrations in gingival crevicular fluid from patients with periodontitis compared with periodontally healthy individuals. Higher Gal-3 levels have also been described in more advanced periodontal disease, including stage III grade C periodontitis, suggesting that local Gal-3 expression may reflect the severity of periodontal inflammatory activity [40].

Mechanistically, increased Gal-3 expression in periodontal lesions may contribute to macrophage recruitment, polarization toward a profibrotic phenotype, osteoclast activation, matrix metalloproteinase activity, and replacement of normal periodontal structures by inflamed or fibrotic granulation tissue. These local processes are relevant because they place Gal-3 at the interface between inflammation, tissue destruction, and extracellular-matrix remodeling within the periodontal compartment [38,40].

In the cardiovascular system, Gal-3 is widely recognized as a marker and mediator of myocardial fibrosis. Activated macrophages are an important source of Gal-3 in fibrotic cardiac tissue, and Gal-3 can stimulate fibroblast activation, collagen production, and maladaptive remodeling through pathways that interact with TGF-β-related signaling and other inflammatory mediators [39,41]. Elevated circulating Gal-3 concentrations have been associated with heart failure severity, adverse prognosis, and more active myocardial remodeling, supporting its role as a systemic indicator of inflammatory–fibrotic activity [39].

Gal-3 has also been investigated in relation to atrial fibrillation. Patients with AF have been reported to show higher serum Gal-3 concentrations than individuals in sinus rhythm, and higher Gal-3 levels have been described in persistent or chronic AF compared with paroxysmal AF [39]. In patients undergoing AF ablation, increased pre-procedural Gal-3 concentrations have been associated with a higher risk of arrhythmia recurrence, suggesting that Gal-3 may reflect a residual fibrotic substrate that persists despite rhythm-control intervention [42,43]. Clinical and mechanistic literature further supports this relationship, linking Gal-3 to inflammatory–fibrotic atrial remodeling rather than to a purely electrical AF substrate [13,39,42,43,44].

Consistent with this fibrotic role, a prospective study of patients with paroxysmal AF found that higher baseline plasma Gal-3 concentrations independently predicted progression to persistent AF over follow-up, supporting Gal-3 as a marker of advancing atrial structural remodeling rather than a static risk indicator alone [44].

The connection between periodontal Gal-3 activity and atrial remodeling remains biologically plausible but incompletely demonstrated. Chronic periodontal inflammation could contribute to systemic Gal-3 activation, thereby reinforcing a profibrotic cardiovascular milieu. In support of this concept, higher salivary and serum Gal-3 levels have been observed when periodontal disease coexists with systemic inflammatory comorbidities such as chronic kidney disease, suggesting that local and systemic inflammatory burdens may interact [12]. However, studies directly correlating periodontal-tissue Gal-3 expression with circulating Gal-3 concentrations, atrial structural remodeling, or AF burden remain limited.

Overall, Gal-3 represents the strongest clinical biomarker candidate among the pathways reviewed here. Its increased expression in periodontal inflammatory environments and its association with myocardial fibrosis, atrial remodeling, AF progression, and post-ablation recurrence support its relevance within the periodontitis–AF axis. Nevertheless, available evidence remains largely compartmentalized, with periodontal, circulating, and cardiovascular Gal-3 measurements usually assessed separately. Therefore, Gal-3 should be regarded as a promising bridging biomarker, while its value as an integrated tissue-to-cardiovascular marker requires further clarification.

### 3.6. SGLT2-Related Pathways and Potential Modulation of AF Risk

Sodium–glucose cotransporter 2 (SGLT2) is expressed predominantly in the renal proximal tubules, where it mediates most filtered glucose reabsorption. In contrast, SGLT2 expression in healthy human myocardium and vascular endothelium appears to be minimal or absent, whereas SGLT1 and facilitative glucose transporters are more relevant for myocardial glucose handling [45]. Therefore, the cardiovascular effects of SGLT2 inhibitors are unlikely to result primarily from direct blockade of a myocardial SGLT2 receptor. Instead, their benefits are generally interpreted as indirect and systemic, involving renal, hemodynamic, metabolic, inflammatory, oxidative-stress, and antifibrotic mechanisms [45].

Although initially developed as antihyperglycemic agents, SGLT2 inhibitors have shown pleiotropic effects relevant to cardiovascular remodeling. Clinical and experimental data indicate that these drugs may reduce circulating inflammatory mediators, including IL-6, TNF-α, and high-sensitivity C-reactive protein, and may attenuate pro-inflammatory signaling through pathways involving NF-κB and the NLRP3 inflammasome [46]. Additional effects include reduced oxidative stress, modulation of macrophage polarization, improvement in endothelial function, and attenuation of profibrotic signaling. These mechanisms provide a plausible explanation for the observed cardiovascular benefits of SGLT2 inhibition beyond glycemic control [46].

In relation to atrial fibrillation, accumulated evidence suggests that SGLT2 inhibitors may reduce the risk of incident AF in patients with cardiometabolic disease, heart failure, or chronic kidney disease. A meta-analysis of randomized clinical trials reported a lower risk of new-onset AF among patients treated with SGLT2 inhibitors compared with placebo [15]. A subsequent trial-level meta-analysis including a larger randomized population also found an overall reduction in incident AF, although the effect appeared more evident in patients with heart failure with reduced ejection fraction and less consistent in populations with mildly reduced or preserved ejection fraction [47]. These findings suggest that the antiarrhythmic effect of SGLT2 inhibition may depend on the degree and type of underlying structural and cardiometabolic remodeling.

Several mechanisms may contribute to the observed association between SGLT2 inhibition and reduced AF risk. Natriuretic and diuretic effects may decrease volume overload and atrial filling pressures, thereby limiting atrial dilatation. Improvements in diastolic function, systemic inflammation, oxidative stress, epicardial adipose-tissue activity, and myocardial fibrosis may also reduce the development of an arrhythmogenic atrial substrate [15,46,47]. In patients with established AF, observational data have suggested lower recurrence rates after catheter ablation among diabetic patients receiving SGLT2 inhibitors, but this evidence remains non-randomized and should be interpreted cautiously [48].

More recent evidence suggests that SGLT2 inhibitor use may be associated with a lower rate of AF recurrence after catheter ablation in patients with type 2 diabetes or heart failure, although available data remain partly observational and population-dependent [49]. In patients with chronic kidney disease, the protective effect appears less uniform and may depend on sample size, follow-up duration, and the specific agent used [50]. In addition, recent evidence in older adults with AF suggests that coexisting periodontitis may modify the relationship between diabetes and clinical outcomes, supporting the need to consider periodontal status within broader cardiometabolic risk assessment [51].

The relationship between SGLT2-related pathways and periodontal disease is less well established. Diabetes mellitus and periodontitis interact bidirectionally: poor glycemic control may worsen periodontal inflammation, while chronic periodontal inflammation may contribute to insulin resistance and systemic inflammatory activation. Improved glycemic control has been associated with more favorable periodontal parameters, supporting the metabolic component of periodontal disease severity [52]. Limited clinical observations and case-based evidence suggest that SGLT2 inhibition may be accompanied by improvement in some periodontal or infection-related outcomes in selected populations, but large clinical trials specifically evaluating periodontal endpoints are lacking [53].

Direct evidence linking periodontal inflammation with SGLT2-axis modulation remains limited. We did not identify clinical studies showing that periodontal therapy modifies SGLT2 expression or SGLT2-related signaling in humans. However, periodontal treatment has been reported to improve periodontal parameters and modulate local and systemic inflammatory profiles, supporting the broader concept that reducing periodontal inflammatory burden may influence systemic metabolic and inflammatory pathways [54]. Preclinical evidence provides additional biological plausibility: in a diabetic mouse model, exposure to *Porphyromonas gingivalis* lipopolysaccharide increased renal SGLT2 gene and protein expression compared with diabetic controls, suggesting that periodontal pathogen-derived inflammatory stimuli may influence SGLT2 expression under diabetic conditions [14]. Whether similar mechanisms occur in humans with periodontitis remains unknown.

In summary, SGLT2-related pathways should be interpreted as potential modifiers of the systemic cardiometabolic and inflammatory environment rather than as direct periodontal tissue biomarkers comparable to TSP-1 or Gal-3. The available evidence supports a plausible association between SGLT2 inhibition, reduced systemic inflammation, attenuated fibrotic remodeling, and lower AF risk in selected cardiometabolic populations. However, the specific periodontitis–SGLT2–AF axis remains largely indirect and hypothesis-generating. Its relevance may be greatest in patients in whom periodontal inflammation coexists with diabetes, heart failure, chronic kidney disease, or other cardiometabolic conditions that amplify systemic inflammatory–fibrotic activity.

### 3.7. Effects of Periodontal Treatment on Inflammation and AF-Related Outcomes

Periodontal treatment is relevant to the periodontitis–AF hypothesis because it may reduce the local inflammatory burden that contributes to systemic cytokine activation, endothelial dysfunction, and profibrotic signaling. Intensive periodontal interventions, including scaling, root planing, professional debridement, and hygiene optimization, have been associated with reductions in circulating inflammatory mediators such as CRP, IL-6, and TNF-α in patients with periodontal disease [54]. These findings support the concept that periodontal inflammation is not only locally expressed but may also contribute to a modifiable systemic inflammatory profile.

The potential relevance of periodontal therapy to AF-related outcomes has been examined in patients undergoing rhythm-control interventions. In a prospective clinical study, patients with periodontitis received periodontal treatment shortly after catheter ablation for AF and were followed for approximately one year. Among patients with severe periodontitis, periodontal treatment was associated with a markedly lower risk of AF recurrence compared with the absence of post-ablation periodontal therapy. In addition, patients who experienced AF recurrence had higher periodontal inflamed surface area values than those who maintained sinus rhythm [7]. These findings suggest that periodontal inflammatory burden may influence the post-ablation arrhythmogenic substrate.

Preventive oral-care patterns may also be relevant to long-term AF risk. In an observational analysis, regular use of dental services, including professional cleaning and prophylactic care, was associated with a lower long-term risk of incident AF compared with less consistent dental-care use [6]. Although this association may reflect both better periodontal control and broader differences in health behavior, it supports the possibility that sustained oral-health maintenance may reduce systemic inflammatory burden over time.

Despite these observations, the available interventional evidence remains insufficient to establish that periodontal treatment directly prevents AF onset or recurrence. Existing studies are limited by observational designs, selected populations, modest sample sizes, and potential confounding by metabolic status, cardiovascular risk factors, medication use, oral-health behavior, and access to healthcare. Moreover, most studies have not simultaneously assessed periodontal tissue markers, circulating inflammatory mediators, cardiac imaging indices, and rhythm-monitoring outcomes.

Periodontal treatment may therefore represent a biologically plausible modifier of systemic inflammation and AF-related risk, particularly in patients with active periodontal inflammation and established cardiovascular vulnerability. However, its role should currently be interpreted as supportive and hypothesis-generating rather than as a proven antiarrhythmic intervention. The available evidence reinforces the need to determine whether changes in local periodontal inflammatory activity are paralleled by changes in systemic biomarkers and cardiovascular remodeling.

### 3.8. Integrated Synthesis and Remaining Mechanistic Uncertainty

The evidence reviewed above supports a multilevel inflammatory–fibrotic framework linking periodontal disease with cardiovascular and atrial remodeling. At the local level, periodontitis is characterized by dysbiotic biofilm, epithelial ulceration, immune-cell recruitment, cytokine release, and extracellular-matrix remodeling. These processes may extend beyond the periodontal compartment through recurrent bacteremia, dissemination of microbial products, systemic cytokine activation, endothelial dysfunction, oxidative stress, and thromboinflammatory signaling [21,22,23,24].

At the clinical level, observational studies have associated periodontal disease with cardiovascular phenotypes such as atherosclerotic disease, hypertension, heart failure, and atrial fibrillation [3,6,23,25,26,27,28]. Among these outcomes, AF is particularly relevant because its pathophysiology depends not only on electrical triggers but also on atrial structural remodeling, inflammation, fibrosis, endothelial dysfunction, and cardiometabolic stress. Human atrial tissue data further strengthen the biological plausibility of this relationship, as periodontal inflammatory burden has been associated with atrial fibrosis, non-paroxysmal AF, and left atrial appendage thrombosis in selected patients with established AF [29,30].

Among the molecular mediators discussed in this review, TSP-1 and Gal-3 appear especially relevant because they are implicated in both periodontal inflammatory environments and cardiovascular fibrotic remodeling. TSP-1 may connect periodontal immune activation with endothelial dysfunction and TGF-β-related collagen deposition [10,33,35,36,37], while Gal-3 may link macrophage activation, extracellular-matrix remodeling, and atrial fibrotic substrate formation [12,13,39,42,43,44]. However, the strength of evidence differs between these markers: Gal-3 has more extensive clinical evidence in AF and post-ablation recurrence [13,39,42,43,44], whereas TSP-1 remains supported mainly by mechanistic, periodontal-tissue, and selected cardiovascular data [10,33,35,36,37].

SGLT2-related pathways occupy a different position within this framework. Unlike TSP-1 and Gal-3, SGLT2 should not be interpreted as a direct periodontal tissue biomarker. Instead, SGLT2 inhibition may modify the systemic cardiometabolic, inflammatory, oxidative-stress, hemodynamic, and antifibrotic environment in which both periodontal disease and AF develop [15,47,48,49,50]. The available evidence supports a possible relationship between SGLT2 inhibition and lower AF risk, but the specific link between periodontal inflammation, SGLT2-axis modulation, and atrial remodeling remains indirect and hypothesis-generating [14].

Despite the coherence of this model, several uncertainties remain. Most available studies evaluate only one compartment of the proposed pathway: periodontal clinical status, circulating biomarkers, cardiovascular outcomes, or atrial tissue remodeling. Far fewer studies integrate these levels simultaneously. As a result, it remains unclear whether molecular changes within periodontal tissue reflect systemic inflammatory–fibrotic activity, whether they parallel circulating concentrations of TSP-1 or Gal-3, or whether they correspond to atrial remodeling severity, AF phenotype, or arrhythmia burden.

Therefore, the current evidence supports a biologically plausible but incompletely characterized continuum: periodontal inflammation may contribute to systemic inflammatory activation, which may in turn promote endothelial dysfunction, profibrotic signaling, atrial remodeling, and AF susceptibility. However, causality, temporal sequence, tissue concordance, and the clinical utility of biomarker-based risk stratification remain insufficiently established (Table 1). This unresolved mechanistic space is central to the interpretation of the periodontitis–AF relationship.

## 4. Discussion

This structured narrative review supports a plausible inflammatory–fibrotic framework linking periodontitis with atrial remodeling and AF, but the strength of evidence is uneven across the pathway. The most consistent clinical evidence concerns the association between periodontitis and AF risk and the association between Gal-3 and atrial fibrosis or AF recurrence. Evidence for TSP-1/TGF-β signaling is mainly mechanistic and tissue-level, while the periodontitis–SGLT2–AF connection remains indirect.

A central finding of this synthesis is that the periodontal–AF relationship should be interpreted through the concept of atrial cardiomyopathy rather than through electrical arrhythmogenesis alone. AF development and persistence depend on structural remodeling, interstitial fibrosis, conduction heterogeneity, endothelial dysfunction, inflammation, autonomic influences, and cardiometabolic stress. Within this framework, chronic periodontal inflammation may contribute to the systemic environment that favors atrial remodeling, although the available evidence does not establish periodontitis as an independent causal determinant of AF.

Cardiometabolic studies provide useful background but should not distract from the central periodontitis–AF question. Data linking triglyceride-to-HDL cholesterol ratio, metabolic syndrome, psoriatic arthritis, insulin resistance, and endothelial dysfunction to AF-related phenotypes support the broader inflammatory and metabolic context in which AF develops [17,18,19]. However, these findings should be interpreted as contextual support rather than as direct evidence for a periodontal origin of atrial remodeling.

Human tissue data are particularly relevant because they provide a closer link between periodontal inflammatory burden and the atrial substrate. Associations between periodontal indices, periodontal inflamed surface area, left atrial appendage fibrosis, non-paroxysmal AF, and atrial thrombus suggest that periodontal inflammation may be related to more advanced structural and thromboinflammatory atrial phenotypes [29,30]. However, these studies were conducted in selected patients with established AF and surgical indications; therefore, they cannot clarify whether periodontal inflammation precedes atrial remodeling, develops in parallel with it, or reflects shared cardiometabolic risk. Complementary imaging evidence also supports the relevance of a combined inflammatory and structural substrate in AF: in a coronary computed tomography study, AF was associated with higher epicardial adipose tissue volume, higher left atrial volume index, and increased fat attenuation index, suggesting that adipose-tissue inflammation and atrial enlargement may be integrated non-invasive markers of AF-related remodeling [55]. In this context, multimodal cardiac imaging, including transthoracic echocardiography, transesophageal echocardiography, computed tomography, and cardiac magnetic resonance, may provide complementary information on left atrial and left atrial appendage pathology, cardiac sources of embolism, and structural substrates relevant to AF-related cardioembolic risk [56].

Among the molecular mediators reviewed, Gal-3 currently has the strongest clinical association with AF, atrial remodeling, and post-ablation recurrence. Its role in macrophage activation, fibroblast stimulation, collagen deposition, and extracellular-matrix remodeling makes it a relevant marker of the inflammation–fibrosis axis [39,41,42,43]. TSP-1 is supported by more mechanistic and tissue-level evidence, particularly through its local expression in periodontal inflammation, its interaction with TGF-β signaling, and its role in endothelial dysfunction and fibrotic remodeling [10,35,36,37]. Together, Gal-3 and TSP-1 provide a biologically coherent link between periodontal tissue inflammation and cardiovascular fibrotic pathways, although direct concordance between periodontal-tissue expression, circulating concentrations, and atrial remodeling remains insufficiently established.

SGLT2-related pathways occupy a different position in this model. Unlike Gal-3 and TSP-1, SGLT2 should not be considered a direct periodontal or atrial tissue biomarker. Instead, SGLT2 inhibition may influence the systemic cardiometabolic environment through renal, hemodynamic, metabolic, anti-inflammatory, antioxidant, and antifibrotic effects [45,46]. The reported association between SGLT2 inhibitor therapy and lower AF incidence or recurrence is clinically relevant, especially in patients with diabetes, heart failure, chronic kidney disease, or cardiometabolic risk [15,47,48]. However, the specific relationship between periodontal inflammation, SGLT2-axis modulation, and atrial remodeling remains indirect and should be regarded as hypothesis-generating [14].

From a clinical perspective, the available evidence supports closer attention to periodontal health in patients with cardiovascular risk factors and AF vulnerability. Periodontal treatment may reduce local and systemic inflammatory burden, and limited evidence suggests a possible association with reduced AF recurrence after ablation [7,54]. Nevertheless, current data do not justify presenting periodontal therapy as a proven antiarrhythmic intervention. A more cautious interpretation is that periodontal assessment and treatment may represent one component of integrated risk-factor management, particularly in patients with active periodontal inflammation and cardiometabolic comorbidity.

This review also highlights an important methodological limitation in the current literature. Most studies evaluate periodontal disease, circulating biomarkers, cardiac imaging, atrial histology, or rhythm outcomes separately. As a result, the relationship between local periodontal molecular activity and systemic or cardiac inflammatory–fibrotic remodeling remains incompletely characterized. This gap is especially relevant for markers such as TSP-1 and Gal-3, which are detectable in periodontal inflammatory environments and are also implicated in cardiovascular fibrosis. Whether periodontal molecular signatures mirror systemic biomarker activity or atrial remodeling severity remains an unresolved question.

Future studies should move beyond isolated associations and integrate periodontal, circulating, imaging, and rhythm-monitoring data within the same patient populations. A key priority is to determine whether the intensity of periodontal inflammatory burden parallels systemic inflammatory–fibrotic activity, left atrial enlargement, atrial fibrosis, and AF burden. Studies combining standardized periodontal evaluation with circulating biomarkers, echocardiographic, CT-based, or cardiac magnetic resonance indices of atrial remodeling, rhythm monitoring, and, where available, tissue-level inflammatory markers could help clarify whether periodontitis is merely a comorbidity associated with AF or part of a broader inflammatory–fibrotic phenotype [29,30,55,56].

From a molecular perspective, Gal-3 and TSP-1/TGF-β-related signaling appear to be the most relevant candidate pathways for further evaluation. Gal-3 has stronger clinical evidence linking it to myocardial fibrosis, AF phenotype, and post-ablation recurrence [13,39,42,43,44], whereas TSP-1 provides a mechanistically plausible connection between periodontal inflammation, endothelial dysfunction, TGF-β activation, and extracellular-matrix remodeling [10,33,35,36,37]. The recent biomarker and molecular-profiling literature also supports the importance of studying fibrotic regulators, galectin biology, and tissue-level cardiovascular transcriptomic signature in a more integrated way [57,58,59]. Future investigations should therefore examine whether these biomarkers, assessed locally and systemically, identify patients with more advanced cardiovascular remodeling or higher AF vulnerability.

Interventional studies are also needed, but they should be designed cautiously. Periodontal treatment has been associated with reductions in systemic inflammatory mediators and with lower AF recurrence after ablation in selected populations, but current evidence remains insufficient to establish periodontal therapy as an antiarrhythmic intervention [7,54]. Future trials should evaluate whether improvement of periodontal inflammation is accompanied by measurable changes in circulating inflammatory biomarkers, atrial remodeling parameters, and rhythm outcomes.

Finally, SGLT2-related pathways require separate consideration. SGLT2 inhibitors may reduce AF risk through systemic cardiometabolic, anti-inflammatory, hemodynamic, oxidative-stress, and antifibrotic mechanisms, particularly in patients with diabetes, heart failure, or chronic kidney disease [15,45,46,47,48]. However, the specific relationship between periodontal inflammation, SGLT2-axis modulation, and atrial remodeling remains indirect. Future studies should therefore treat SGLT2 inhibition primarily as a potential modifier of the systemic cardiometabolic environment rather than as a direct periodontal biomarker [14].

Overall, future research should aim to clarify temporal sequence, tissue-level concordance, and clinical relevance. The key unresolved question is whether molecular inflammatory–fibrotic activity in periodontal tissues reflects systemic biomarker activation and cardiovascular remodeling, or whether these phenomena simply coexist because of shared cardiometabolic risk factors.

Several limitations of the present review should be acknowledged. First, the article is a structured narrative review rather than a systematic review; therefore, evidence selection was purposive and thematic rather than exhaustive. Second, the included literature is heterogeneous in terms of periodontal definitions, cardiovascular endpoints, biomarker assays, AF phenotype classification, follow-up duration, and adjustment for confounders. Third, much of the evidence remains observational, and residual confounding by age, diabetes, obesity, smoking, socioeconomic status, oral-health behavior, medication use, and access to healthcare cannot be excluded. Finally, the mechanistic link between periodontal tissue inflammation and atrial remodeling remains incompletely defined, particularly in human studies integrating periodontal, circulating, imaging, and rhythm-monitoring data.

Overall, the periodontitis–AF relationship appears best understood as part of a broader inflammatory–fibrotic and cardiometabolic continuum, rather than as a single linear causal pathway. Periodontal inflammation may contribute to systemic immune activation and endothelial dysfunction, while Gal-3, TSP-1/TGF-β signaling, and SGLT2-related pathways represent complementary but unequally supported molecular perspectives on fibrosis, inflammation, and cardiometabolic modulation (Table 2). The available evidence is promising but still insufficient to establish causality or to define biomarker-based clinical pathways. The most important unresolved issue is whether local periodontal molecular changes parallel systemic inflammatory–fibrotic activity and clinically relevant cardiovascular remodeling.

## 5. Conclusions

Periodontitis and atrial fibrillation appear to share a biologically plausible inflammatory–fibrotic background involving systemic immune activation, endothelial dysfunction, oxidative stress, thrombo-inflammatory signaling, and extracellular-matrix remodeling. Within this framework, periodontal inflammation may contribute to a systemic environment that favors atrial structural remodeling and AF susceptibility, although current evidence does not establish a direct causal relationship.

Among the molecular pathways reviewed, Gal-3 has the strongest clinical association with atrial fibrosis, AF burden, and post-ablation recurrence, whereas TSP-1/TGF-β provides a mechanistic bridge between periodontal inflammation, endothelial dysfunction, and fibrotic remodeling. SGLT2-related pathways should be interpreted separately as indirect systemic modifiers of cardiometabolic, inflammatory, oxidative-stress, and profibrotic environments rather than as indirect periodontal or atrial tissue biomarkers.

Future studies should clarify temporal sequence, causality, tissue-level concordance, and the extent to which local periodontal molecular changes reflect systemic or cardiac inflammatory–fibrotic activity. Integrated assessment of periodontal status, circulating biomarkers, cardiac imaging, and rhythm monitoring may help refine interdisciplinary cardiovascular risk assessment and identify more precise inflammatory and fibrotic markers relevant to AF vulnerability.

## 6. Take-Home Message

In patients with elevated cardiovascular risk, particularly those with atrial fibrillation or evidence of atrial remodeling, evaluation and appropriate treatment of periodontitis may represent a reasonable component of integrated risk-factor management. However, current evidence does not establish that periodontal treatment directly prevents atrial fibrillation, and the molecular relationship between periodontal inflammation and atrial remodeling remains incompletely defined.

## Figures and Tables

**Table 1:** Representative clinical, imaging, biomarker, and experimental evidences relevant to the periodontitis-AF inflammatory-fibrotic continuum.

Study/Evidence Domain	Population or Model	Design	Main Finding Relevant to This Review	Limitations/Evidence Strength
Park et al. [6]	>1.25 million individuals without baseline AF	Nationwide population-based cohort	Persistent periodontitis was associated with higher incident AF; improvement of periodontal status was associated with slightly lower risk than persistent disease.	Large clinical association; residual confounding and healthcare-behavior bias remain possible.
Sen et al. [27]; Leelaviwat et al. [28]; Xin-Nian and Xin-Yi [31]	Community cohorts and pooled observational studies	Cohort study and systematic reviews/meta-analyses	Periodontal disease was consistently associated with higher AF or atrial flutter risk across available observational evidence.	Supports association; does not establish temporality, causality, or tissue-level mechanisms.
Miyauchi et al. [29,30]	76 patients with established AF undergoing left atrial appendage excision	Human histological study	Periodontal inflammatory indices were associated with atrial fibrosis, non-paroxysmal AF, and left atrial appendage thrombus.	Closer tissue-level evidence, but selected surgical AF population and cross-sectional interpretation.
Miyauchi et al. [9]; Xiang et al. [32]	Human atrial specimens, murine periodontitis model, and transcriptomic/immune-cell datasets	Translational and experimental studies	*P. gingivalis* translocation and shared immune-genetic signatures were linked to atrial fibrosis and Gal-3/TGF-β1 or RGS1-related mechanisms.	Mechanistically informative; human validation and temporal sequence remain limited.
Gal-3 biomarker studies [39,42,43,44]	Patients with AF, ablation cohorts, and biomarker meta-analysis	Clinical biomarker studies and meta-analysis	Gal-3 was associated with myocardial fibrosis, AF phenotype, progression, and post-ablation recurrence.	Stronger clinical evidence for AF/fibrosis, but usually not measured simultaneously with periodontal tissue markers.
SGLT2 inhibitor evidence [15,47,48,49,50]	Patients with diabetes, heart failure, chronic kidney disease, or post-ablation AF risk	Randomized-trial meta-analyses and observational studies	SGLT2 inhibitor therapy was associated with lower incident AF or post-ablation AF recurrence in selected cardiometabolic populations.	Indirect for periodontitis–AF; should be interpreted as systemic cardiometabolic modulation, not direct periodontal evidence.
Gerculy et al. [55]	122 patients undergoing coronary computed tomography; 42 with AF and 80 without AF	Retrospective cardiac CT imaging study	AF was associated with higher epicardial adipose tissue volume, left atrial volume index, and fat attenuation index, supporting a combined structural and inflammatory imaging substrate.	Human imaging support for inflammatory/structural substrate; not a periodontal study and does not prove causality.

**Table 2:** Candidate molecular links and hierarchy of evidence between periodontal inflammation, atrial remodeling, and atrial fibrillation.

Pathway/Marker	Current Evidence Level	Key Periodontal and Cardiovascular Relevance	Interpretation
TSP-1/TGF-β	Mechanistic/periodontal-tissue/selected cardiovascular evidence	Gingival inflammation, local matrix remodeling, endothelial dysfunction, TGF-β activation, and atrial fibrosis in selected cardiovascular settings.	Plausible bridging pathway; not yet an established causal mediator
Gal-3	Stronger clinical biomarker evidence	Periodontal inflammatory activity, myocardial fibrosis, AF phenotype, AF progression, and post-ablation recurrence.	Most clinically supported bridging biomarker, but periodontal–circulating–atrial concordance remains uncertain
IL-6/TNF-α/CRP	Non-specific inflammatory evidence	Systemic inflammatory spillover from active periodontal lesions; endothelial dysfunction, oxidative stress, AF risk, and recurrence.	Important inflammatory background, but low specificity for periodontal origin.
SGLT2-related pathways	Indirect/hypothesis-generating for the periodontitis–AF connection	Mainly metabolic and inflammatory interaction in diabetes/periodontitis; lower AF risk or recurrence with SGLT2 inhibitors in selected cardiometabolic populations	Systemic modifier, not a direct periodontal or atrial tissue biomarker.

## Data Availability

No new data were created or analyzed in this study. Data sharing is not applicable to this article.
